# Role of bevacizumab in the prevention of early postoperative haemorrhage after 25-gauge microincision vitrectomy surgery

**DOI:** 10.12669/pjms.325.10362

**Published:** 2016

**Authors:** Zaheer Sultan, Syed Fawad Rizvi, Faisal Murtaza Qureshi, Syed Asaad Mahmood

**Affiliations:** 1 Dr. Zaheer Sultan, FCPS. LRBT Free Base Eye Hospital, Korangi 2 ½, Karachi, Pakistan; 2 Dr. Syed Fawad Rizvi, FCPS. LRBT Free Base Eye Hospital, Korangi 2 ½, Karachi, Pakistan; 3 Dr. Faisal Murtaza Qureshi, FRCS. LRBT Free Base Eye Hospital, Korangi 2 ½, Karachi, Pakistan; 4 Dr. Syed Asaad Mahmood, MBBS. LRBT Free Base Eye Hospital, Korangi 2 ½, Karachi, Pakistan

**Keywords:** Bevacizumab, Diabetic retinopathy, Micro incision vitrectomy surgery, Prognosis, Vitreous haemorrhage

## Abstract

**Objective:**

To evaluate the effect of preoperative intravitreal bevacizumab injection on the incidence of postoperative haemorrhage and visual prognosis, in patients undergoing 25-gauge micro incision vitrectomy surgery (MIVS) for diabetic vitreous haemorrhage.

**Methods:**

One hundred and twenty two eyes of 122 patients of diabetic retinopathy of both genders and aged over 18 years, who presented with non-resolving vitreous haemorrhage were enrolled for this study. All patients received an intravitreal injection of 1.25 mg/0.05 mL bevacizumab (Avastin) which was followed one week later by 25-gauge sutureless micro incision vitrectomy surgery. Main outcomes measured were best corrected visual acuity (BCVA) assessed with logMAR and post-operative vitreous haemorrhage. Follow ups were up to six months post-operatively. IBM SPSS 21 was used for data analysis.

**Result:**

A total of 122 patients were included; 78 (63.9%) males and 44 (36.1%) females. Mean age at the time of surgery was 51.4 ± 13.66 years. The mean preoperative BCVA was 1.64 ± 0.427 logMAR which improved to 0.57 ± 0.253 logMAR at 12 months post-operatively (p-value < 0.001). Recurrent vitreous haemorrhage was seen in four patients (3.28%).

one1 week before 25-gauge vitrectomy helps to reduce the incidence of early post-vitrectomy haemorrhage in diabetic patients.

## INTRODUCTION

The prevalence of proliferative diabetic retinopathy (PDR) among diabetic patients in Pakistan is estimated to be around 27.43%.^1^,^2^ Pan retinal photocoagulation (PRP) remains the primary treatment for PDR, but once complications occur, such as vitreous haemorrhage or tractional retinal detachment, vitrectomy surgery is advocated.^3^,^4^ In 1975, O’Malley developed the first 20-gauge 3-port pars plana vitrectomy (PPV).^5^ The instruments and technology involved in this procedure has seen a significant progression over the last few decades and transconjunctival suture less 25-gauge micro incision vitrectomy system (MIVS) are in common use today. Compared to the earlier PPV, MIVS facilitates early visual recovery while decreasing patient discomfort, operating time, post-operative inflammation, ocular surface irregularities and surgically induced astigmatism.^6^-^8^

The late 1990’s saw a continuous improvement of surgical technique which resulted in significantly improved outcomes in vitrectomies performed for diabetic retinopathy.^9^ Numerous medications and surgical techniques were tested out with the aim to prevent vitreous haemorrhage, out of which intravitreal bevacizumab was found to be efficacious. Bevacizumab (Avastin, Genentech Inc., San Francisco, CA), is a humanized monoclonal antibody against vascular endothelial growth factor (VEGF) that is thought to induce regression of retinal neovascularization and enhance the clearance of vitreous haemorrhage.^10^ It has been suggested as an adjunctive therapy with laser photocoagulation and as intravitreal injection before vitrectomy to decrease the risk of early bleeding.^2^,^11^ However, the optimal timing of intravitreal bevacizumab (IVB) prior to vitrectomy is debateable.

As with any other surgical procedure, there are some associated complications. The most common of these is recurrent vitreous haemorrhage which may occur in up to 60% of eyes in the postoperative period.^12^ Haemorrhage typically happens within a week of surgery but it could present months later. In cases of severe PDR, tractional retinal detachment has been seen to develop following intravitreal bevacizumab injections.^13^

The rationale of this study was to evaluate the effect of preoperative intravitreal bevacizumab injection on the incidence of postoperative haemorrhage in patients with proliferative diabetic retinopathy (PDR), undergoing 25-gauge micro incision vitrectomy surgery (MIVS) for diabetic vitreous haemorrhage. Secondary outcomes measures included changes in best-corrected visual acuity (BCVA) and IVB-related adverse events.

## METHODS

After obtaining the hospital ethics committee approval, this prospective, non-comparative, interventional study was started at LRBT tertiary eye hospital Karachi in January 2013. Patients were admitted until December 2014 using non probability convenience sampling technique and the data collected from follow ups visits up to June 2015.

One hundred and twenty two eyes of 122 patients with diabetic retinopathy, aged over 18 years of both genders, who presented with non-resolving vitreous haemorrhage were included. Excluded from these were patients with concomitant funnel shaped retinal detachment, whether it was evident on B-scan during pre-operative evaluation or if was revealed per-operatively. Moreover, patients with glaucoma, uveitis, cataract, history of previous posterior segment ocular surgery, history of intravitreal injections and those following up for less than 12 months were excluded from the analysis.

Patient demographics, preoperative and postoperative visual acuity, intraocular pressure, slit lamp examination details, indirect ophthalmoscopy findings, density of vitreous haemorrhage, presence of retinal detachment, and any postoperative complications experienced was recorded on a proforma. Systemic assessment included blood pressure measurement and HbA1c. For consistency, all the pre-operative values used in analysis were recorded one week before the MIVS procedure, so as to exclude any effect of intravitreal injection on the data. The entire procedure along with its risks and benefits was explained to the patient and a written informed consent was taken.

An intravitreal injection of 1.25 mg/0.05 mL bevacizumab (Avastin, Genentech) was given in the supero-temporal quadrant via a30-gauge needle through the pars plana in the operating theatre, in the standard sterile method under topical anaesthesia. The patient was discharged with atopical prophylactic antibiotic (moxifloxacin) for three days. These patients then underwent 25-gauge 3-port vitrectomy (MIVS), one week (6-8 days) after the intravitreal injection. All patients were operated by a single vitreo-retinal surgeon using a Constellation vitrectomy system (Alcon Laboratories Inc.) under local anaesthesia. After complete vitrectomy, and removal of retinal traction, pan retinal photocoagulation was done using the endolaser probe.

Postoperative follow ups were at one day, one week, one month, three months, and six months. Best corrected visual acuity was measured using a Snellen chart at 6 metres by an experienced optometrist, which was converted to logMAR notation for analysis. Any complications found during the follow up visits were noted down in the proforma and managed accordingly. Statistical data was analysed on IBM SPSS Statistics 21 using paired sample t- test with 95% confidence interval, where applicable. A p-value of < 0.05 was considered statistically significant.

## RESULTS

One hundred and twenty two eyes of 122 patients were included in this study. Seventy eight (63.9%) patients were males and 44 (36.1%) were females. Mean age at the time of surgery was 51.4± 13.66 years. The mean preoperative BCVA was 1.64± 0.427 logMAR which improved to 0.57± 0.253 logMAR at 12 months post-operatively (p-value < 0.001). Pre-operative IOP was 16.1 ± 4.46mmHg which came down to 15.4 ±3.99 mmHg after surgery (p-value = 0.23). No injection related complications were observed. No intravitreal injections were given post-operatively during the follow-up period. Complications encountered during and after the surgery are shown in Table-I.

**Table-I:** Complications.

	*Eyes (n=122)*	*Percentage*
Intraoperative bleed	10	8.19%
Post-op Vitreous Hg	4	3.28%
Cystoid macular edema	19	15.57%
Retinal re-detachment	1	0.82%
Endophthalmitis	0	0
Cataract	38	31.14%
Transient hypotony	8	6.56%
IOP > 22 mmHg	3	2.46%
ERM	3	2.46%

## DISCUSSION

The role of IVB in reducing vitreous haemorrhage from retinal neovascularization secondary to diabetic retinopathy has been evaluated in a number of studies. This effect is thought to be a result of reduction of leakage from the foci of neovascularization, as evident on fluorescein angiography, and regression of the neovascular component of fibrovascular tissue.^2^,^14^ Some studies have also demonstrated IVB to be effective in the resolution of vitreous haemorrhage. This led researchers to believe that IVB may play a role in reducing intraoperative and postoperative haemorrhage in diabetic vitrectomy.

Recurrent vitreous haemorrhage after primary vitrectomy has been reported in up to 80% of eyes over a six month period.^15^ Since the postoperative haemorrhage usually occurs within the first few days after surgery, the VEGF blockade provided by a single dose of bevacizumab for four weeks is sufficient to prevent postoperative bleeding in the vast majority of cases.^12^ This was observed in this study with very low incidence of post-operative vitreous haemorrhage. Other studies which have compared the preoperative IVB group against a control MIVS-only group have reported approximately a three times increased risk of intraoperative bleeding when performing MIVS alone.^16^ A 2015 Cochrane review concluded that IVB resulted in the occurrence of postoperative vitreous haemorrhage in 512 fewer people per 1000 people treated as compared to people undergoing pars plana vitrectomy alone.

Ishikawa et al assessed the efficacy of IVB given 3 to 30 days before vitrectomy and reported a reduction in bleeding during surgical dissection of fibrovascular membranes.^17^ Lauro et al. compared the efficacy of preoperative bevacizumab administered 20 days before vitrectomy with bevacizumab seven days preoperatively and found better surgical results in the latter group.^18^ Furthermore, there have been reports of IVB leading to worsening of tractional retinal detachments and Pokroy et al. limiting IVB to 2–10 days before surgery.^9^

The role of bevacizumab in preventing postoperative vitreous haemorrhage after vitrectomy for diabetic eye disease is also reinforced through a study by Cheema et al. where bevacizumab injected at the end of surgery reduced recurrent postoperative vitreous haemorrhage to 4%, compared with 42% in the control group.^19^

The mean duration of surgery in this study was 28.2± 4.21 minutes. However, the surgeries were performed by a single vitreoretinal surgeon who has an expertise of over 10 years with the Micro-incision vitrectomy system. It is possible that for less experienced surgeons, avoiding intra-operative bleeds may have a significant improvement on the total surgery time by providing a clearer view during surgery. The reduction in surgical time in patients receiving IVB before vitrectomy has also been reported by several authors.^16^,^20^,^21^ This is the consequence of less instrumentation requirement, clearer view during vitrectomy, reduced risk of creating iatrogenic breaks and easier dissection of membranes and decreased need of endolaser.^12^

Among other advantages of preoperative IVB that have been reported in literature, a systemic review by Smith et al. found some evidence of a lower risk of postoperative retinal detachment in the participants treated with pre- or intraoperative bevacizumab.^22^ A study by Guthrie et al. suggests improves laser uptake, easier fibrovascular membrane dissection, and decreases silicon oil use as advantages of preoperative IVB^23^. Some studies have reported a better final best corrected visual acuity (BCVA) in patients receiving IVB before undergoing MIVS for PDR as compared to the control group.^24^,^25^ However, other similar studies did not find any statistically significant difference between the groups for postoperative BCVA.^23^

The most common complication encountered in this study was cataract development in phakic eyes. Postoperative cataract progression and hypotony are generally the most common postoperative complications reported in literature for eyes undergoing 25-gauge MIVS.^26^

### Limitations of the Study

While this study shows promising results for pre-operative IVB in a relatively larger sample of patients than that of similar studies carried out previously, the lack of a control group was a limitation in this study, and therefore the extent of improvement over patients undergoing MIVS alone could not be judged statistically.

## CONCLUSION

Intravitreal bevacizumab (1.25 mg/0.05 mL) one week prior to MIVS for diabetic vitreous haemorrhage was helpful in reducing the intra- and post-operative bleeding. Consequently, it results in a quicker surgery and a better mean post-operative visual acuity.
